# Synchrotron X‐Ray Fluorescence Nanoprobe Reveals Target Sites for Organo‐Osmium Complex in Human Ovarian Cancer Cells

**DOI:** 10.1002/chem.201605911

**Published:** 2017-01-26

**Authors:** Carlos Sanchez‐Cano, Isolda Romero‐Canelón, Yang Yang, Ian J. Hands‐Portman, Sylvain Bohic, Peter Cloetens, Peter J. Sadler

**Affiliations:** ^1^ Department of ChemistryUniversity of WarwickCoventryCV4 7ALUK; ^2^ ID16A beamline, ESRFThe European Synchrotron71 Avenue des Martyrs3800GrenobleFrance; ^3^ Inserm, U836, equipe 6, “Rayonnement synchrotron et recherches medicales”, GrenobleInstitut des Neurosciences38054GrenobleFrance; ^4^ School of Life SciencesUniversity of WarwickCoventryCV4 7ALUK

**Keywords:** bioinorganic chemistry, elemental mapping, metallodrugs, organometallic complexes, X-ray fluorescence

## Abstract

A variety of transition metal complexes exhibit anticancer activity, but their target sites in cells need to be identified and mechanisms of action elucidated. Here, it was found that the sub‐cellular distribution of [Os(η^6^‐*p*‐cym)(Azpy‐NMe_2_)I]^+^ (*p*‐cym=*p*‐cymene, Azpy‐NMe_2_=2‐(*p*‐[dimethylamino]phenylazo)pyridine) (**1**), a promising drug candidate, can be mapped in human ovarian cancer cells at pharmacological concentrations using a synchrotron X‐ray fluorescence nanoprobe (SXRFN). SXRFN data for Os, Zn, Ca, and P, as well as TEM and ICP analysis of mitochondrial fractions suggest localization of Os in mitochondria and not in the nucleus, accompanied by mobilization of Ca from the endoplasmic reticulum, a signaling event for cell death. These data are consistent with the ability of **1** to induce rapid bursts of reactive oxygen species and especially superoxide formed in the first step of O_2_ reduction in mitochondria. Such metabolic targeting differs from the action of Pt drugs, offering promise for combatting Pt resistance, which is a current clinical problem.

The most widely used drugs in cancer chemotherapy are platinum complexes, cisplatin, carboplatin, and oxaliplatin. Despite their success, resistance to platinum is now a clinical problem, as is the need to reduce side‐effects and extend treatment to a wider range of cancers.^1^ These challenges might be remedied by complexes of other second and third‐row transition metals if they have different mechanisms of action from platinum, for instance by targeting sites other than nuclear DNA or by causing distinctly different lesions on DNA. Recently, organometallic half‐sandwich Os^II^ arene complexes have shown promise.^2^ Using phenotypic screening, we discovered high activity within a series of Os^II^ arene azopyridine complexes. For example, [Os(η^6^‐*p*‐cym)(Azpy‐NMe_2_)I]PF_6_ (where *p*‐cym=*p*‐cymene and Azpy‐NMe_2_=2‐(*p*‐[dimethylamino]phenylazo)pyridine) (**1**, Scheme 1) is 49× more potent than cisplatin towards a range of 809 cancer cell lines, and not cross‐resistant with cisplatin or oxaliplatin.^3^ Identifying the target sites for metallodrugs in cells is a challenging task, but is assisted for precious metals by their normal absence.

**Scheme 1:**
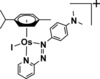
Structure of [Os(η^6^‐*p*‐cym)(Azpy‐NMe_2_)I]^+^ (**1**).

Two promising techniques for characterizing the sub‐cellular distribution of metallodrugs are secondary ion mass spectrometry (SIMS) and synchrotron‐based X‐ray fluorescence (SXRF). Importantly, these high resolution multi‐element analysis techniques allow simultaneous mapping of essential elements such as P and Zn in cells. The recent development of nanoscale probing for SIMS (NanoSIMS) has allowed cellular organelles to be analyzed. For example, Pt from cisplatin and Triplatin C has been detected by NanoSIMS (maximum resolution of 80×80 nm^2^) in the nucleolus of MCF7 cells,^4^ whereas Ru from antimetastatic organometallic complexes has been found in membranes or intercellular junctions,^5^ and gold phosphine anticancer complexes in the nucleus and cytoplasm.^6^ However, NanoSIMS offers low sensitivity towards second and third‐row transition metals (due to low ionization yield),^7^ and requires treatment of cells with elevated drug concentrations.

SXRF has also been used to investigate anticancer metal compounds in vivo and in vitro. Thus, the cellular distribution of Pt, Gd, Ru, Se, and As metallodrugs^8^ and Nb, Co, Ti, and V metallocenes has been explored by micro‐SXRF.^9^ Pt‐based DNA‐intercalators have been found concentrated in the nuclei of cells, whereas cisplatin is evenly distributed throughout the cell.8c SXRF experiments have suggested that Gd complexes carrying triphenylphosphonium substituents can localize in the mitochondria of cells (although mitochondria were not distinctly resolved in the maps acquired)8h and that Zn plays an important role in the resistance to cisplatin.8b However, the spatial resolution achieved previously by micro‐SXRF (from 0.2×0.2 to 1.5×1.5 μm^2^ beam size) made it difficult to assess accurately the subcellular localization of metals, especially within small organelles with sizes ≤1 μm (which cannot be resolved). It also usually required treatment of cells with extremely high concentrations of drug (>100 μm), although more relevant concentrations have been used in some experiments (1–20 μm).8b,8d,8i,8j

Newly designed SXRF nanoprobes (SXRFN) now allow acquisition of elemental maps with sub‐100×100 nm^2^ resolution.^10^ For example, SXRFN analysis revealed the localization of a Ru antimalarial drug within the digestive vacuole of *Plasmodium falciparum*‐infected erythrocytes (80×80 nm^2^ spot size; scanned using a 100×100 nm^2^ step size).10a

Here we use the new nano‐imaging beamline ID16A at the ESRF, capable of focussing the hard X‐ray beam size down to 27×37 nm^2^. This represents a significant reduction in the surface of the spot size compared to previous use, and provides sufficient resolution for the study of sub‐cellular organelles. Furthermore, the unique flux of about 4×10^11^ photons per second available at ID16A allows detection of organometallic anticancer complexes at medically relevant concentrations in thin cellular sections.^11^

We acquired SXRFN elemental maps for A2870 human ovarian carcinoma cells treated with **1**, using an excitation energy of 17.05 keV and a beam‐size of 27×37 nm^2^ (H×V) in all experiments. Samples were scanned using step sizes of 50×50 nm^2^ or 20×20 nm^2^ (the smallest reported for biological samples). This sampling is consistent with the resolution offered by the focus (≈2 pixel elements per resolution element). Sample preparation is important for the preservation of biologically relevant elements in adherent mammalian cells. Initial maps showed that paraformaldehyde (PFA) preserved the structure of A2780 cells better than methanol fixation (Figure S1 in the Supporting Information). Interestingly, recent reports suggest that PFA fixation is also not ideal; although it maintains the localization of Zn, it leads to increased elemental leaching when compared to PFA+glutaraldehyde fixation or vitrification followed by freeze drying.^12^ The appropriate level of exposure of cells to **1** so as to achieve intracellular SXRFN detection of Os was optimized using ICP‐MS (Table S1). As a result, Os maps for whole A2780 cells treated for 24 h with biologically relevant concentrations of **1** (IC_50_: 0.16 μm or 6× IC_50_: 1 μm) and fixed with 2 % PFA were acquired using a 50×50 nm^2^ scan step size and 50 ms dwell time (Figure 1 and Figure S2 in the Supporting Information). These maps confirmed that **1** is detectable even at drug concentrations as low as 160 nm. SXRFN maps acquired from 500 nm‐thick sections of Epon‐embedded cells (1 μm **1**, 24 h; raster scan step sizes 50×50 nm^2^ or 20×20 nm^2^; 50 ms dwell time) showed that **1** readily penetrates cells (Figures 2 and 3 and Figures S4 and S5 in the Supporting Information). Osmium localization does not overlap with Zn and there is only marginal overlap with P (Figures 2 and 3; confirmed by co‐localization analysis, Figures S6 and S7). Zinc and P are found mostly in cell nuclei (e.g., as Zn‐finger proteins).^13^ This indicates that **1** is not localized in the nuclei of treated cells, and suggests that DNA is not a major target for **1**.

**Figure 1:**
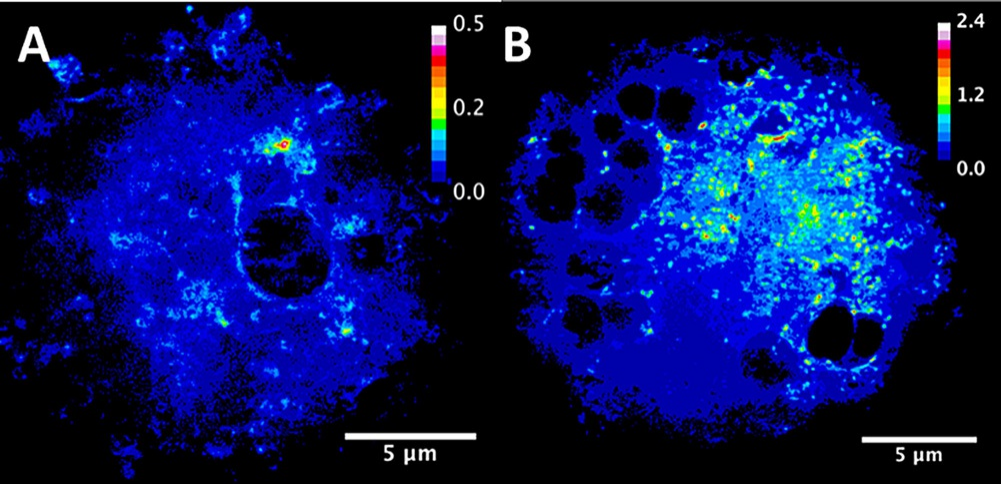
XRF maps of Os in whole A2780 ovarian cancer cells treated for 24 h with A) IC_50_ concentration of **1**; B) 1 μm **1**. Raster scan: 50×50 nm^2^ step size, 50 ms dwell time. Scale bar 5 μm. Calibration bar in ng mm^−2^.

**Figure 2:**
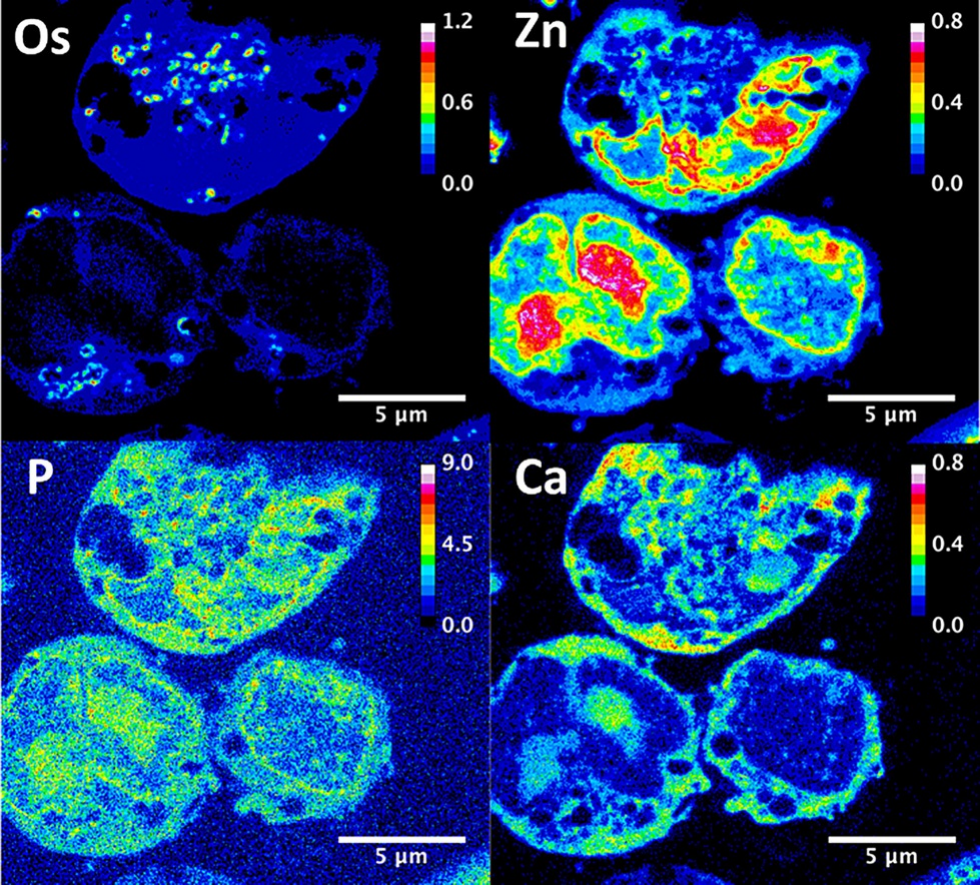
XRF maps of a 500 nm thick section of A2780 cells treated for 24 h with 1 μm **1** showing the cellular distribution of Os, Zn, P, and Ca. Raster scan: 50×50 nm^2^ step size, 50 ms dwell time. Scale bar 2 μm. Calibration bar in ng mm^−2^.

**Figure 3:**
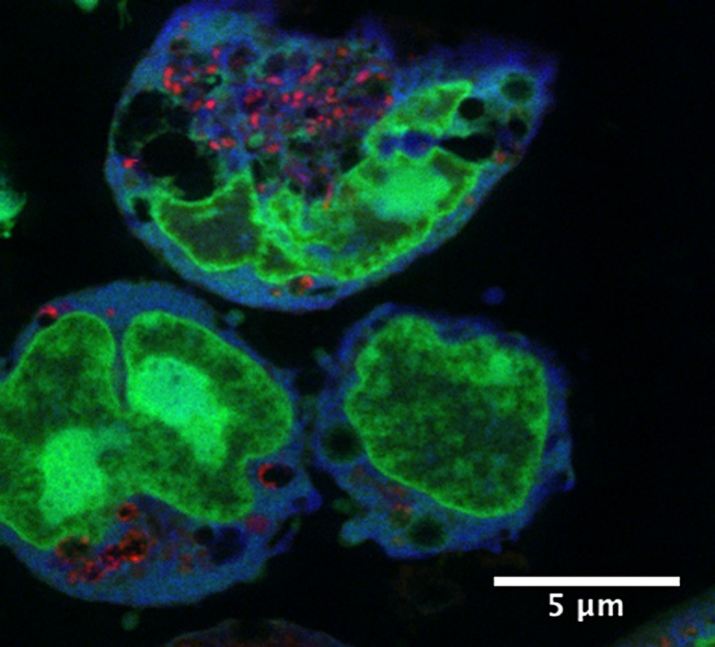
Co‐localization XRF map of a 500 nm‐thick section of A2780 cells treated for 24 h with 1 μm **1** showing the cellular distribution of Os (red), Zn (green), and Ca (blue). Raster scan: 50×50 nm^2^ step size, 50 ms dwell time. Scale bar 5 μm.

Interestingly, Os from **1** appeared to be significantly compartmentalized within small organelles in cells, concentrated in small elliptical areas approximately 270 nm in length (Figures 1–4 and Figure S8 in the Supporting Information). SXRFN maps of 3T3 cells with mitochondria labeled with gold have shown similar patterns.10c Additionally, TEM images showed the existence of dark particles of around 350 nm in length in treated and 400 nm in untreated cells (Figure S9). These elliptical particles are similar in size and shape to the mitochondria of fast‐growing tumor cells such as A2780, and to other organelles such as endosomes, lysosomes, and peroxisomes. Attempts to identify organelle localization of **1** using Os–Au co‐localization in SXRFN maps with antibody‐conjugated nanogold^TM [14]^ were unsuccessful due to the low efficiency of antibody labeling (data not shown).

**Figure 4:**
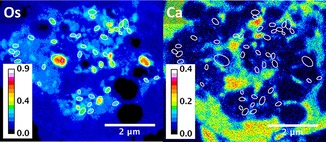
XRF maps of a 500 nm‐thick section of A2780 cells treated for 24 h with 1 μm **1** showing the cellular distribution of Os and Ca. Raster scan: 20×20 nm^2^ step size, 50 ms dwell time. White ellipses show the absence of Ca in areas with high density of Os. Scale bar 2 μm. Calibration bar in ng mm^−2^.

Supporting evidence for localization of Os in mitochondria was obtained by isolating mitochondrial fractions from A2780 cells and determining their Os content by ICP‐MS. This increased from (4±1) to (49±7) ng Os per milligram protein when the treatment dose was increased from 0.16 μm to 1 μm **1** (Figure S10), whereas no Os was found in the mitochondria of untreated cells.

Additionally, SXRFN mapping showed that treatment of A2780 cells with **1** leads to significant perturbations in the cellular localization of bound Ca (Figures 2 and 3 and Figure S5). Calcium in cells is stored in the endoplasmic reticulum (ER). Intracellular levels of “free” Ca are normally very low (ca. 100 nm) and mobilization of stored Ca^2+^ is a signalling event leading to allosteric regulation of proteins and enzymes. High levels of cytoplasmic Ca^2+^ can stimulate mitochondrial activity and play a key role in apoptosis (programmed cell death), by activating the caspase chain after mitochondrial overload, as well as other types of cell death processes such as necrosis or autophagy.^15^

Free Ca^2+^ normally leaks out of cells after fixation, so only Ca chelated by proteins or other biomolecules can be detected. Our elemental maps show that bound Ca is concentrated in the nucleus in untreated A2780 cells, as it correlates well with Zn and P (Figure S5 in the Supporting Information). However, upon treatment of cells with 1 μm of **1** we detect only extranuclear Ca, which is spread over the cytoplasm. Furthermore, Zn maps and TEM images show dramatic fragmentation of the nucleus and membrane blebbing (Figures 2 and 3 and Figure S9). Interestingly, cell‐cycle analysis of A2780 treated with 1 μm **1**, shows S‐G2/M arrest and abnormally high sub‐G0 populations (Figure S11), but Ca is not found in areas with a high density of Os, suggesting that mitochondrial loading with Ca might have not occurred (at least not after 24 h; Figure 4 and Figure S12, highlighted areas).

Complex **1** is relatively inert in chemical reactions but appears to be activated by glutathione in cancer cells resulting in initial formation of the hydroxido complex and loss of the iodido ligand, which is rapidly pumped out (Figure 5).^16^ Both the intact complex and hydroxido metabolite are lipophilic cations, species often readily taken up by mitochondria.^17^ We have observed rapid bursts of reactive oxygen species (ROS) and especially superoxide on treatment of cancer cells with **1**.^18^ Proteins in the inner membrane of mitochondria are active in electron transport and oxidative phosphorylation and are rich sources of ROS. Moreover, when GSH (an antioxidant) in cancer cells is depleted by l‐buthionine sulfoximine (l‐BSO), the activity of **1** is enhanced several‐fold.^17^ Additionally, Ca homeostasis is controlled by cellular levels of ROS, and high levels of radicals can induce the release of Ca from the ER to the cytoplasm.^19^

**Figure 5:**
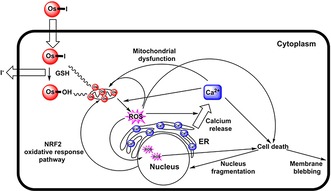
Possible pathways that link intracellular activation of **1** with Ca mobilization, mitochondrial dysfunction, ROS generation, and cell death.

Complex **1** is approximately 49× more active than cisplatin over a range of 809 cancer cell lines. RNA sequencing and proteomic analysis of A2780 cells treated with **1** have shown a dramatic effect on the transcription of the mitochondrial chromosome indicative of mitochondrial dysfunction, together with a fast cellular response involving a mitochondria‐linked oxidative stress response pathway (NRF2), and downregulation of key genes, consistent with ROS production and reduction in the ability of cells to respond.3b The ability of **1** to induce the release of bound Ca from the nucleus to the cytoplasm, and cause nuclear fragmentation as well as membrane blebbing can all be related to mitochondrial damage. Mitochondrial targeting offers a promising new strategy for the design of anticancer complexes. The mitochondria of cancer cells are known to be defective compared to the mitochondria of normal cells. Indeed we have detected three mutations in the mitochondrial DNA of A2780 human ovarian cells, all located in complex **1** (NADH dehydrogenase) of the electron transport chain.3b This difference between normal cells and cancer cells should provide a basis for selective drug activity towards cancer cells and reduce unwanted side‐effects.

These experiments show that with recent advances in the design of synchrotron X‐ray fluorescence nanoprobes it is now possible to map the distribution of metallodrugs in small organelles in cancer cells at pharmacologically relevant concentrations, down to nanomolar Os concentrations. This demonstration of mitochondrial targeting in ovarian cancer cells, together with our previous molecular cellular studies,3b show that complex **1** has an unusual mode of action.

## Experimental Section

XRF experiments were performed on the ID16A beamline at the ESRF synchrotron light source. Irradiation of the samples was done at 17.05 keV energy and detection was performed using a pair of 6‐element silicon drift diode detectors (Sensortech, UK). Scan step size was fixed at 400×400 nm^2^ (dwell time 100 ms) for coarse scans and 50×50 nm^2^ (dwell time 50 ms) for fine scans (although a sample was also measured using scan step size of 20×20 nm^2^). Spectra were fitted using the free PyMCA software.^20^ Due to overlap between Zn‐kα and Os‐Lα_1_ emissions, fitting of Zn‐kβ emission and deconvolution of the signals were also used to provide quantitative data (Figure S2). The fitting procedure includes an attenuation correction that is significant for low Z elements such as P. It assumes a sample matrix consisting of 500 nm thick EPON. The quantitative calibration was determined using a thin film X‐ray fluorescence 7‐element reference sample (AXO Dresden GmbH, Table S2).

The Supporting Information contains details of materials used, cell culture and preparation for TEM (Figure S9), X‐ray phase‐contrast (Figure S3), and XRF (spectrum Figure S2; further elemental maps Figures S1, S4, S5, S11 and S12), Os uptake (Table S1), size distribution of Os regions‐of‐interest (ROIs) (Figure S8), mitochondrial uptake (Figure S10), cycle analysis (Figure S11), fluorescence calibration data (Table S2), and image analysis (Figures S6 and S7).

## Supporting information

As a service to our authors and readers, this journal provides supporting information supplied by the authors. Such materials are peer reviewed and may be re‐organized for online delivery, but are not copy‐edited or typeset. Technical support issues arising from supporting information (other than missing files) should be addressed to the authors.

Supplementary

## References

[1] L. Kelland , Nat. Rev. Cancer 2007, 7, 573–584. 17625587 10.1038/nrc2167

[2a] T. C. Johnstone , K. Suntharalingam , S. J. Lippard , Philos. Trans. R. Soc. A 2015, 373, 20140185; 10.1098/rsta.2014.0185 PMC4342973 25666060

[2b] M. Hanif , M. V. Babak , C. G. Hartinger , Drug Discovery Today 2014, 19, 1640–1648. 24955838 10.1016/j.drudis.2014.06.016

[3a] Y. Fu , A. Habtemariam , A. M. Pizarro , S. H. van Rijt , D. J. Healey , P. A. Cooper , S. D. Shnyder , G. J. Clarkson , P. J. Sadler , J. Med. Chem. 2010, 53, 8192–8196; 20977192 10.1021/jm100560f

[3b] J. M. Hearn , I. Romero-Canelón , A. F. Munro , Y. Fu , A. M. Pizarro , M. J. Garnett , U. McDermott , N. O. Carragher , P. J. Sadler , Proc. Natl. Acad. Sci. USA 2015, 112, E3800–E3805. 10.1073/pnas.1500925112 PMC4517206 26162681

[4a] L. E. Wedlock , M. R. Kilburn , R. Liu , J. A. Shaw , S. J. Berners-Price , N. P. Farrell , Chem. Commun. 2013, 49, 6944–6946; 10.1039/c3cc42098a PMC4132055 23687657

[4b] A. A. Legin , A. Schintlmeister , M. A. Jakupec , M. Galanski , I. Lichtscheidl , M. Wagner , B. K. Keppler , Chem. Sci. 2014, 5, 3135–3143. 10.1039/c3sc53426j PMC9273000 35919909

[5] R. F. S. Lee , S. Escrig , M. Croisier , S. Clerc-Rosset , G. W. Knott , A. Meibom , C. A. Davey , K. Johnsson , P. J. Dyson , Chem. Commun. 2015, 51, 16486–16489. 10.1039/c5cc06983a 26426486

[6] L. E. Wedlock , M. R. Kilburn , J. B. Cliff , L. Filgueira , M. Saunders , S. J. Berners-Price , Metallomics 2011, 3, 917–925. 21796317 10.1039/c1mt00053e

[7] P. Hoppe , S. Cohen , A. Meibom , Geostand. Geoanal. Res. 2013, 37, 111–154.

[8a] A. A. Hummer , A. Rompel , Metallomics 2013, 5, 597–614; 23558305 10.1039/c3mt20261e

[8b] M. Shimura , A. Saito , S. Matsuyama , T. Sakuma , Y. Terui , K. Ueno , H. Yumoto , K. Yamauchi , K. Yamamura , H. Mimura , Y. Sano , M. Yabashi , K. Tamasaku , K. Nishio , Y. Nishino , K. Endo , K. Hatake , Y. Mori , Y. Ishizaka , T. Ishikawa , Cancer Res. 2005, 65, 4998–5002; 15958539 10.1158/0008-5472.CAN-05-0373

[8c] K. J. Davis , J. A. Carrall , B. Lai , J. R. Aldrich-Wright , S. F. Ralph , C. T. Dillon , Dalton Trans. 2012, 41, 9417–9426; 22740039 10.1039/c2dt30217a

[8d] M. D. Hall , C. T. Dillon , M. Zhang , P. Beale , Z. Cai , B. Lai , A. J. Stampfl , T. W. Hambley , J. Biol. Inorg. Chem. 2003, 8, 726–732; 12884089 10.1007/s00775-003-0471-6

[8e] M. D. Hall , R. A. Alderden , M. Zhang , P. J. Beale , Z. Cai , B. Lai , A. J. Stampfl , T. W. Hambley , J. Struct. Biol. 2006, 155, 38–44; 16630726 10.1016/j.jsb.2006.01.011

[8f] S. Antony , J. B. Aitken , S. Vogt , B. Lai , T. Brown , L. Spiccia , H. H. Harris , J. Biol. Inorg. Chem. 2013, 18, 845–853; 23943098 10.1007/s00775-013-1027-z

[8g] J. B. Aitken , S. Antony , C. M. Weekley , B. Lai , L. Spiccia , H. H. Harris , Metallomics 2012, 4, 1051–1056; 22907648 10.1039/c2mt20072d

[8h] D. E. Morrison , J. B. Aitken , M. D. de Jonge , J. A. Ioppolo , H. H. Harris , L. M. Rendina , Chem. Commun. 2014, 50, 2252–2254; 10.1039/c3cc46903d 24352097

[8i] E. L. Crossley , J. B. Aitken , S. Vogt , H. H. Harris , L. M. Rendina , Angew. Chem. Int. Ed. 2010, 49, 1231–1233; 10.1002/anie.200902309 20077549

[8j] J. B. Aitken , P. A. Lay , T. T. Hong Duong , R. Aran , P. K. Witting , H. H. Harris , B. Lai , S. Vogt , G. I. Giles , J. Biol. Inorg. Chem. 2012, 17, 589–598; 22327627 10.1007/s00775-012-0879-y

[8k] K. L. Munro , A. Mariana , A. I. Klaving , A. J. Foster , B. Lai , S. Vogt , Z. Cai , H. H. Harris , C. T. Dillon , Chem. Res. Toxicol. 2008, 21, 1760–1769; 18597498 10.1021/tx800128d

[8l] L. E. Wu , A. Levina , H. H. Harris , Z. Cai , B. Lai , S. Vogt , D. E. James , P. A. Lay , Angew. Chem. Int. Ed. 2016, 55, 1742–1745; 10.1002/anie.201509065 26696553

[9] J. B. Waern , H. H. Harries , B. Lai , Z. Cai , M. M. Harding , C. T. Dillon , J. Biol. Inorg. Chem. 2005, 10, 443–452. 15906108 10.1007/s00775-005-0649-1

[10a] F. Dubar , S. Bohic , C. Slomianny , J.-C. Morin , P. Thomas , H. Kalamou , Y. Guerardel , P. Cloetens , J. Khalife , C. Biot , Chem. Commun. 2012, 48, 910–912; 10.1039/c1cc16211j 22143053

[10b] E. L. Que , R. Bleher , F. E. Duncan , B. Y. Kong , S. C. Gleber , S. Vogt , S. A. Garwin , A. R. Bayer , V. P. Dravid , R. K. Woodruff , T. V. O'Halloran , Nat. Chem. 2014, 7, 130–139; 25615666 10.1038/nchem.2133 PMC4315321

[10c] S. Matsuyama , M. Shimura , H. Mimura , M. Fujii , H. Yumoto , Y. Sano , M. Yabashi , Y. Nishino , K. Tamasaku , T. Ishikawa , K. Yamauchi , X-Ray Spectrom. 2009, 38, 89–94;

[10d] S. Gil , A. Carmona , G. Martinez-Criado , A. Leon , Y. Prezado , M. Sabes , Biol. Trace Elem. Res. 2015, 163, 177–183; 25216793 10.1007/s12011-014-0097-2

[10e] B. Laforce , C. Carlier , B. Vekemans , J. Villanova , R. Tucoulou , W. Ceelen , L. Vincze , Sci. Rep. 2016, 6, 29999; 27444797 10.1038/srep29999 PMC4956760

[10f] A. Carmona , P. Cloetens , G. Deves , S. Bohic , R. Ortega , J. Anal. At. Spectrom. 2008, 23, 1083;

[10g] R. Ortega , P. Cloetens , G. Deves , A. Carmona , S. Bohic , PLoS ONE 2007, 2, e925; 10.1371/journal.pone.0000925 PMC1976597 17895967

[10h] S. Chen , J. Deng , Y. Yuan , C. Flachenecker , R. Mak , B. Hornberger , Q. Jin , D. Shu , B. Lai , J. Maser , C. Roehrig , T. Paunesku , S. C. Gleber , D. J. Vine , L. Finney , J. VonOsinski , M. Bolbat , I. Spink , Z. Chen , J. Steele , D. Trapp , J. Irwin , M. Feser , E. Snyder , K. Brister , C. Jacobsen , G. Woloschak , S. Vogt , J. Synchrotron Radiat. 2014, 21, 66–75. 24365918 10.1107/S1600577513029676 PMC3874019

[11] http://www.esrf.eu/UsersAndScience/Experiments/XNP/ID16A.

[12] Q. Jin , T. Paunescu , B. Lai , S.-C. Gleber , S. Chen , L. Finney , D. Vine , S. Vogt , G. Woloschak , C. Jacobsen , J. Microsc. 2017, 265, 81–93. 27580164 10.1111/jmi.12466 PMC5217071

[13] R. McRae , B. Lai , C. J. Fahrni , Metallomics 2013, 5, 52–61. 23212029 10.1039/c2mt20176c PMC3769613

[14] R. McRae , B. Lai , S. Vogt , C. J. Fahrni , J. Struct. Biol. 2006, 155, 22–29. 16473527 10.1016/j.jsb.2005.09.013

[15a] R. Rizzuto , C. Giorgi , A. Romagnoli , P. Pinton , Curr. Mol. Med. 2008, 8, 119–130; 18336292 10.2174/156652408783769571

[15b] G. S. B. Williams , L. Boyman , A. C. Chikando , R. J. Khairallah , W. J. Lederer , Proc. Natl. Acad. Sci. USA 2013, 110, 10479–10486. 23759742 10.1073/pnas.1300410110 PMC3696793

[16] R. J. Needham , C. Sanchez-Cano , X. Zhang , I. Romero-Canelon , A. Habtemariam , M. S. Cooper , L. Meszaros , G. J. Clarkson , P. J. Blower , P. J. Sadler , Angew. Chem. Int. Ed. 2017, 56, 1017–1020; 10.1002/anie.201610290 PMC5412917 28000997

[17] M. P. Murphy , R. A. J. Smith , Annu. Rev. Pharmacol. Toxicol. 2007, 47, 629–656. 17014364 10.1146/annurev.pharmtox.47.120505.105110

[18] I. Romero-Canelón , M. Mos , P. J. Sadler , J. Med. Chem. 2015, 58, 7874–7880. 26397305 10.1021/acs.jmedchem.5b00655 PMC4601049

[19a] G. Ermak , K. J. A. Davies , Mol. Immunol. 2002, 38, 713–721; 11841831 10.1016/s0161-5890(01)00108-0

[19b] R. F. Feissner , J. Skalska , W. E. Gaum , S.-S. Sheu , Front. Biosci. 2009, 14, 1197–1218; 10.2741/3303 PMC2683671 19273125

[19c] A. Görlach , K. Bertram , S. Hudecova , O. Krizanova , Redox Biol. 2015, 6, 260–271. 26296072 10.1016/j.redox.2015.08.010 PMC4556774

[20] V. A. Solé , E. Papillon , M. Cotte , P. Walter , J. Susini , Spectrochim. Acta Part B 2007, 62, 63–68.

