# Supplementary material for: Synchrotron X‐Ray Fluorescence Nanoprobe Reveals Target Sites for Organo‐Osmium Complex in Human Ovarian Cancer Cells
Source: Chemistry. 2017 Jan 26;23(11):2512–6. doi: 10.1002/chem.201605911 (PMC5412901; doi:10.1002/chem.201605911)
Supplement: Supplementary file 1 — Supplementary [file CHEM-23-2512-s001.pdf]

CHEMISTRY
A European Journal

Supporting Information

Synchrotron X-Ray Fluorescence Nanoprobe Reveals Target Sites for Organo-Osmium Complex in Human Ovarian Cancer Cells

Carlos Sanchez-Cano,*[a] Isolda Romero-Canelón,[a] Yang Yang,[b] Ian J. Hands-Portman,[d] Sylvain Bohic,[b, c] Peter Cloetens,*[b] and Peter J. Sadler*[a]

chem_201605911_sm_miscellaneous_information.pdf

Supporting Information

Table of Contents

	**Page**
**Experimental**	**S2**
**Table S1-S2**	**S5**
**Figures S1- S12**	**S6 - S15**

Experimental

Materials. For the biological experiments, RPMI-1640, foetal bovine serum, L-glutamine, penicillin/streptomycin mixture, trypsin/EDTA, and phosphate-buffered saline (PBS) were purchased from GE Healthcare. Propidium iodide (>94%) and RNAse A were obtained from Sigma Aldrich.

Synthesis of [Os(η^6-*p*-cym)(Azpy-NMe$_2$)I]PF$_6$ (1). This complex was synthesized and characterized as described previously.[1]

Cell Culture. A2780 human ovarian carcinoma cells were obtained from the European Collection of Cell Cultures (ECACC), used between passages 5 and 18 and grown in Roswell Park Memorial Institute medium (RPMI-1640) supplemented with 10% v/v of foetal calf serum, 1% v/v of 2 mM glutamine and 1% v/v penicillin/streptomycin. Cells were grown as adherent monolayers at 310 K in a 5% CO_2 humidified atmosphere and passaged at approximately 70-80% confluence.

Metal accumulation in whole cancer cells. Cell accumulation studies for metal complexes were conducted on A2780 human ovarian cells. Briefly, 1 x 10^6 cells were seeded on a 6-well plate. After 24 h of pre-incubation of cells in drug-free medium at 310 K, the complexes were added to give final concentrations equal to IC_{50}, 1 µM and 10 µM, and a further 24 h of drug exposure was then allowed. After this time, cells were washed, treated with trypsin/EDTA, counted, and cell pellets were collected. Each pellet was digested overnight in concentrated nitric acid (73%) at 353 K; the resulting solutions were diluted with a solution containing thiourea and ascorbic acid (to optimise Os speciation for ICP-MS determination)[2] to a final concentration of 5% v/v HNO_3 (final concentration 10 mM thiourea, 0.1 g/L ascorbic acid) and the amount of Os taken up by the cells was determined by ICP-MS (Agilent Technologies 7500 series ICP-MS, detection of ^{189}Os). Data acquisition and analysis was carried out using ICP-MS B.03.05 software). These experiments did not include any cell recovery time in drug-free media; they were carried out in triplicate and the standard deviations were calculated. Os concentrations in cells (in ppm) were calculated using reported values of cell mass and volume.[3]

Preparation of XRF samples (whole cells). Silicon frames with silicon nitride windows (5x5 mm wide, 200 µm thick silicon frames with 1.5x1.5 mm wide and 500

nm thick silicon nitride windows; Silson Ltd) were deposited on 24-well plates and irradiated with UV light for 20 min. Then, A2780 ovarian carcinoma cells were seeded (1×10^5 cells/well) and left to attach for 24 h in RPMI1640 medium. Samples were then treated with IC_{50} or 1 µM of **1**. After 24 h, cells were washed twice with PBS and fixed in methanol at 253 K for 3 min and 30 s exactly, or with 2% PFA at 277 K for 20 min, and then rinsed 3x in PBS, followed by a short wash in ultrapure water (1-2 s). The excess liquid was blotted using pin filter paper and the samples were allowed to dry at room temperature in a clean and dry environment.

Preparation of XRF and TEM samples (sections). A2780 ovarian carcinoma cells were seeded in 100 mm petri dishes (5×10^6 cells/well) and left to attach for 24 h in RPMI1640 medium. Samples were then treated with 1 µM **1**. After 24 h, cells were washed twice with PBS, and fixed for 20 min with 2% glutaraldehyde in sodium cacodylate buffer at pH 7.6 (Agar Scientific) for 1 h, with regular shaking. Then, cells were washed 3x with PBS, and transferred to Falcon tubes using a scrapper prior to centrifugation. Finally, cells were dehydrated with graded levels of ethanol (20-100% ethanol), and then infiltrated with 100% propylene oxide for 1 h followed by a 1:1 mixture of propylene oxide and EPON resin for 6 h. This was replaced with several changes of 100% resin over 18 h before curing for 24 h at 333 K. Blocks were trimmed and sectioned on a Leica Ultracut E ultramicrotome (Leica Microsystems). Sections 500 nm- (for XRF) or 100 nm- (TEM) thick were collected on appropriate sample holders for XRF (5x5 mm wide, 200 µm thick silicon frames with 1.5x1.5 mm-wide and 500 nm-thick silicon nitride windows; Silson Ltd) or TEM (400 mesh Cu grids; sections additionally stained with 2% uranyl acetate), and then imaged according to requirements.

TEM Imaging: Sections prepared for TEM were imaged on Jeol 2010F using a Gatan Ultrascan 4000 camera.

Image Analysis: Images were opened and processed using FIJI ImageJ package[4] with the EDFread plugin. Colocalisation and covariance were calculated selecting the whole cells as ROI and using the Coloc2 plugin.

Metal accumulation in isolated mitochondria. The accumulation of Os from complex **1** in the mitochondrial fraction of A2780 ovarian cells was investigated. Briefly, 20×10^6 cells were seeded on a P145 cell culture dish. After 24 h of pre-

incubation in drug-free medium at 310 K, the complex was added to give final concentrations equal to IC_{50} (0.16 μM) and 1 μM, and a further 24 h of drug exposure was allowed. After this time, cells were washed treated with trypsin/EDTA, and cell pellets were collected. The isolation of mitochondria was carried out using the *Mitochondrial isolation kit for profiling cultured cells* from Sigma Aldrich (MITOISO2), according to the supplier's instructions. Isolated mitochondrial pellets were digested overnight in concentrated nitric acid (73%) at 353 K. The resulting solutions were diluted with a solution containing thiourea and ascorbic acid[2] to a final concentration of 5% v/v HNO_3 (final concentration 10 mM thiourea, 0.1g/L ascorbic acid) and the amount of Os taken up by the cells was determined by ICP-MS as described above. These experiments did not include any cell recovery time in drug-free medium; they were carried out in triplicate and the standard deviations were calculated. The amount of Os determined was normalised against the protein content of each sample determined using the Bradford assay.

Cell cycle analysis. Cells were seeded in a 6-well plate using 1 x 10^6 cells per well. They were pre-incubated in drug-free medium at 310 K for 24 h, after which complex **1** was added at concentrations equal to IC_{50} (0.16 μM) and 1 μM. After 24 h of drug exposure, supernatants were removed by suction and cells were washed with PBS. Finally, cells were harvested using trypsin/EDTA and fixed for 2 h using cold ethanol. DNA staining was achieved by re-suspending the cell pellets in PBS containing propidium iodide (PI) and RNAse A. Cell pellets were re-suspended in PBS before being analysed by flow cytometry using excitation of PI-bound DNA at 536 nm, and emission at 617 nm. Data were processed using Flowjo software. These experiments were carried out in triplicate; although only selected histograms are shown, full numerical data and statistical analysis are below.

References

1. Y. Fu, A. Habtemariam, A. M. Pizarro, S. H. van Rijt, D. J. Healey, P. A. Cooper, S. D. Shnyder, G. J. Clarkson, P. J. Sadler, *J. Med. Chem.* **2010**, 53, 8192-8196.
2. C. Venzago, M. Popp, J. Kovac, A. Kunkel, *J. Anal. At. Spectrom.* **2013**, *28*, 1125-1129.
3. K. Park, J. Jang, D. Irimia, J. Sturgis, J. Lee, J. P. Robinson, M. Toner and R. Bashir, *Lab Chip* **2008**, *8*, 1034-1041.
4. J. Schindelin, I. Arganda-Carreras, E. Frise, V. Kaynig, M. Longair, T. Pietzch, S. Preibisch, C. Rueden, S. Saalfeld, B. Schmid. J.-Y. Tinevez, D.J. White, V. Hartenstein, K. Eliceiri, P. Tomancak and A. Cardona, *Nat. methods* **2012**, *9*, 676.
5. V. A. Solé, E. Papillon, M. Cotte, P. Walter, and J. Susini, *Spectrochim. Acta B* **2007**, *62*, 63-68.

Table S1. Os accumulation in A2780 human ovarian cancer cells treated with various concentrations of complex **1** for different times.

[complex 1] (μM)	10	1	0.16 (IC_{50})
**Exposure Time (h)**	6	24	24
**Os (ppm)**	35.8	230.1	40.3

Table S2. Elemental area density of a 7-element RF7-200-S2371 reference sample (according to the supplier, AXO Dresden GmbH) and the experimental calibration, together with the statistical uncertainties and percentage difference. Molybdenum was not used for the calibration due to the overlap of the Mo Lα and the Pb Mα fluorescence emissions at an excitation energy of 17.05 keV. The calibration was done on a sum spectrum corresponding to 1054 sampling points and a total integration time of 105 s.

Element	Emission Lines	Supplier value (ng/mm^2)	Experimental value (ng/mm^2)	Difference (%)
**Ca**	K	11.4±5.5	11.23±0.006	-1.5
**Fe**	K	4±0.4	3.66±0.002	-8.4
**Cu**	K	2.4±0.5	2.06±0.001	-14
**Pd**	L	1.9±0.8	3.02±0.009	59
**La**	L	9±1.9	12.01±0.005	33
**Pb**	L	7.7±1.3	6.46±0.001	-16

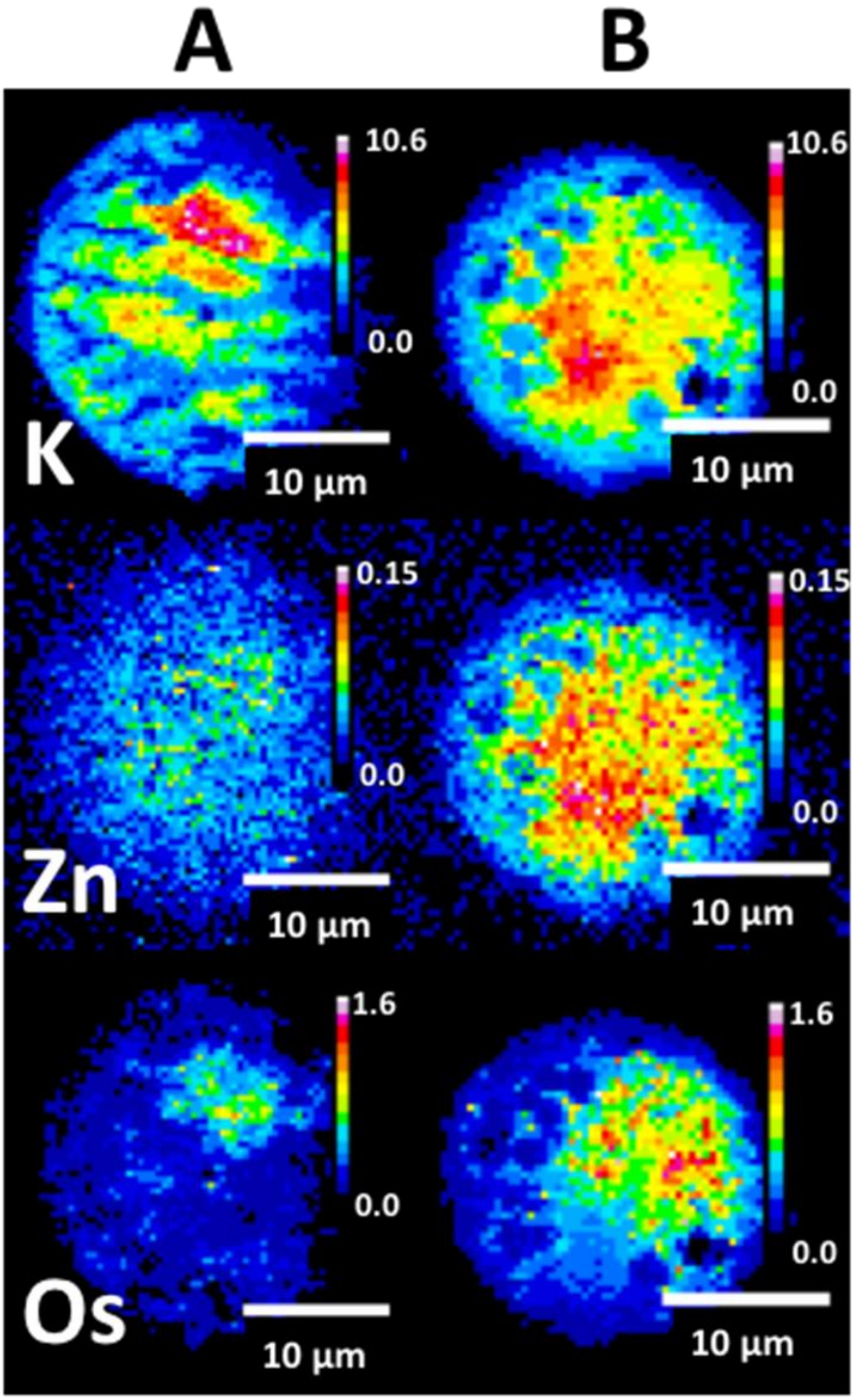

Figure S1. XRF maps of K, Zn and Os in A2780 ovarian cancer cells treated with 1 µM **1** for 24 h and fixed with A) methanol, or B) PFA. Raster scan: 400x400 nm^2 step size, 100 ms dwell time. Scale bar 10 µm. Calibration bar in ng/mm^2.

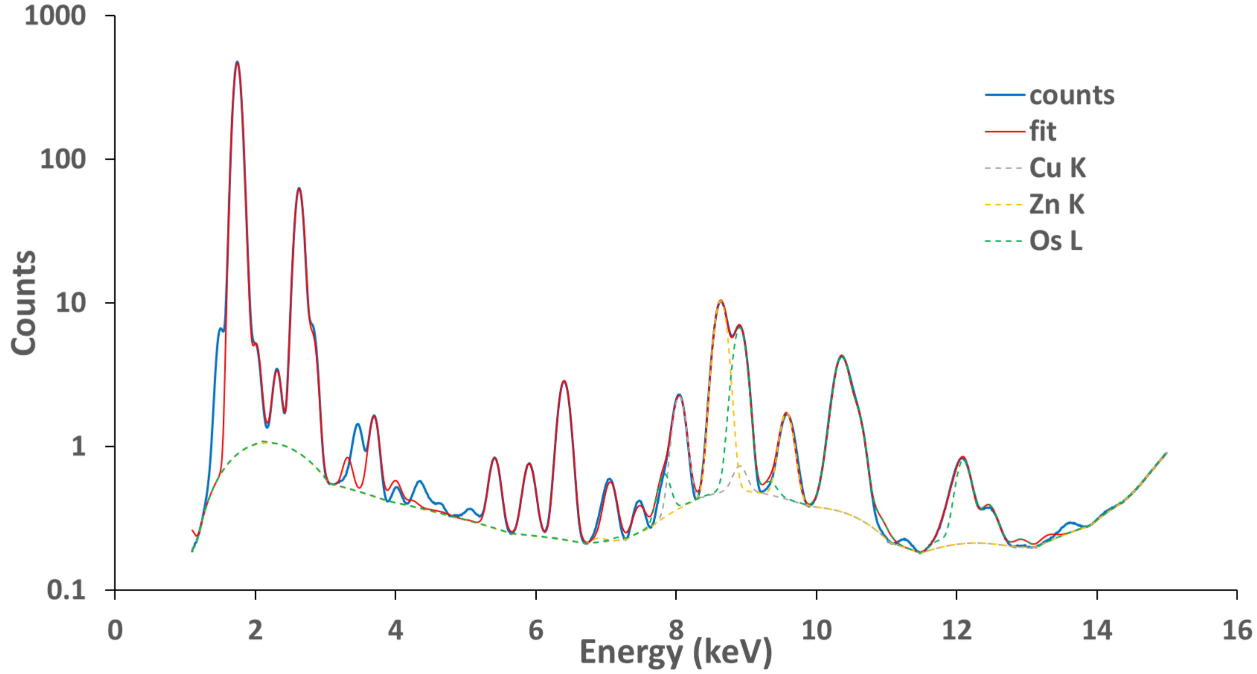

Figure S2. Normalised XRF spectrum from a whole A2780 cell treated with 0.16 µM (IC_{50}) of complex **1** for 24 h; Raster scan: 50x50 nm^2 step size, 50 ms dwell time. The spectrum was fitted using PyMca,[5] and the contribution of selected elements is displayed: Cu and Zn K x-ray emission lines, and Os L X-ray emission lines.

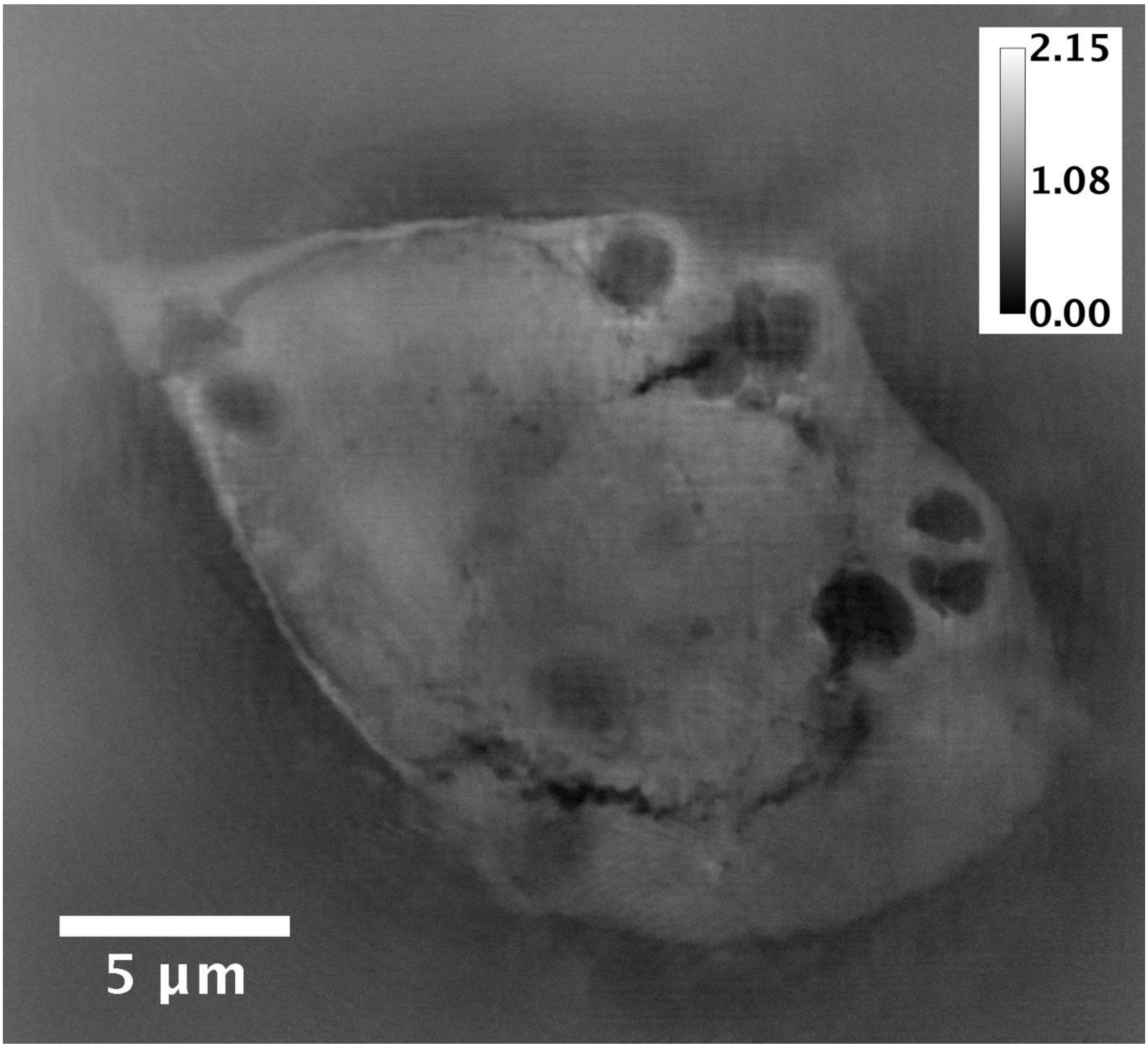

Figure S3. Phase map of a whole A2780 cell treated with 0.16 µM (IC_{50}) of complex **1** for 24 h. Scale bar 5 µm. Calibration bar in µg/mm^2.

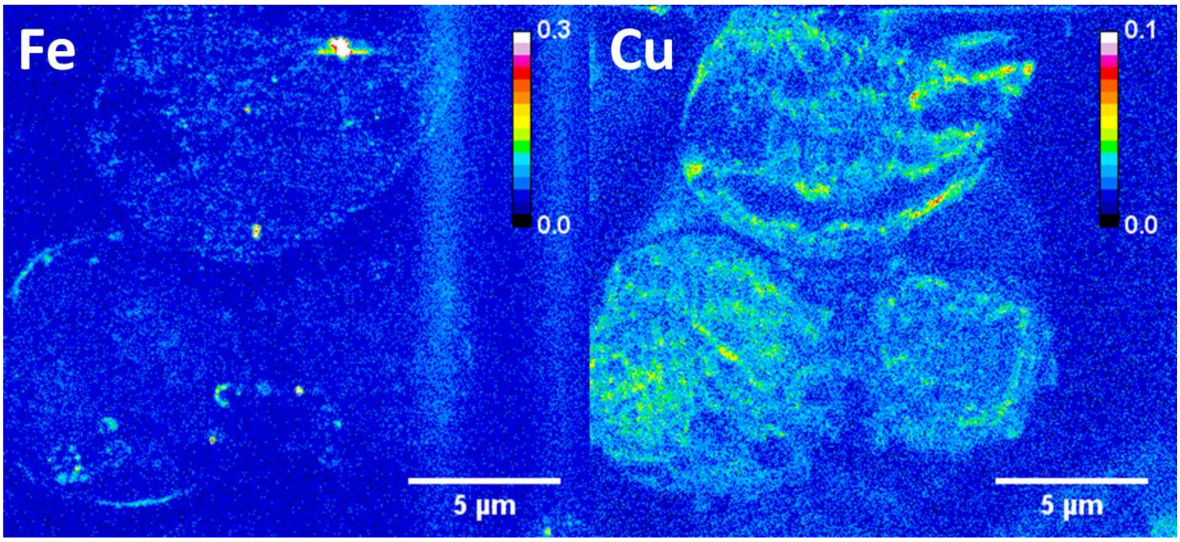

Figure S4. XRF maps of a 500 nm thick section of A2780 cells treated with 1 µM **1** for 24 h showing the distribution of Fe and Cu; Raster scan: 50x50 nm^2 step size, 50 ms dwell time. Scale bar 5 µm. Calibration bar in ng/mm^2.

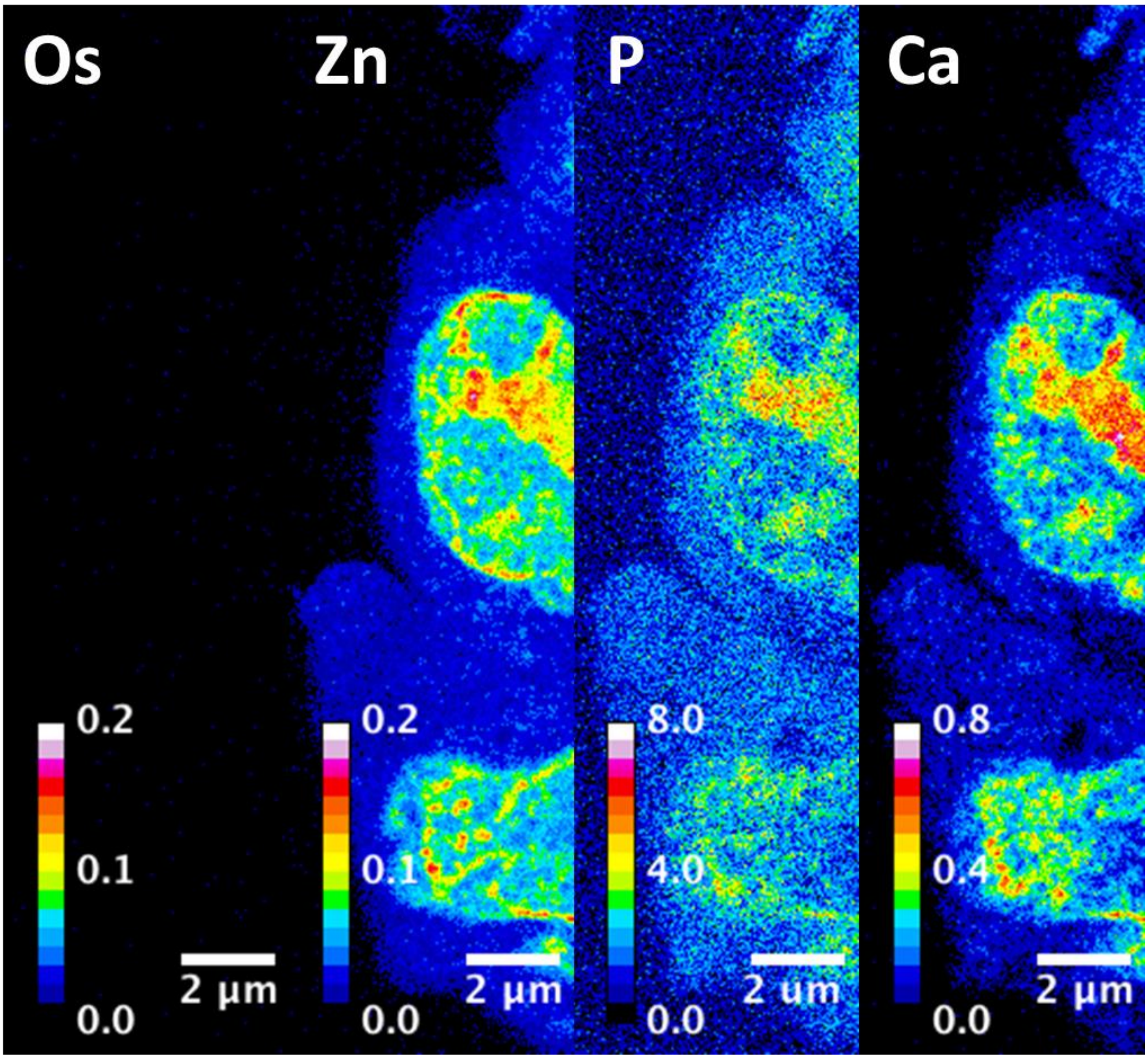

Figure S5. XRF maps of a 500 nm thick section of untreated A2780 cells showing the cellular distribution of Os, Zn, P and Ca; Raster scan: 50x50 nm^2 step size, 50 ms dwell time. Scale bar 2 µm. Calibration bar in ng/mm^2.

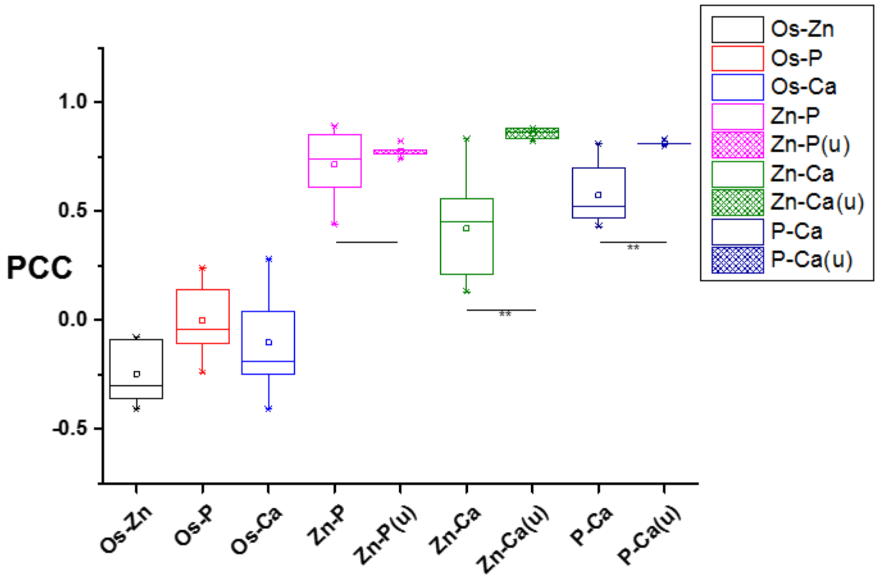

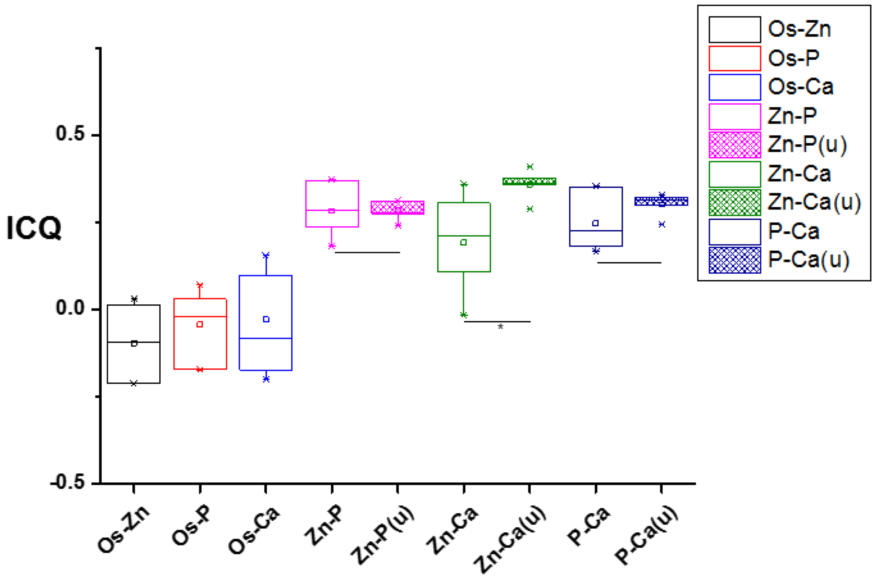

Figure S6. Colocalization analysis for A2780 human ovarian cancer cells treated for 24 h with 1 μM **1,** and for untreated cells (u) between different elements analysed by SXRFN maps using Pearson's correlation coefficient (PCC; +1 perfect colocalization, -1 perfect anti-colocalization; top), and the intensity correlation quotient (ICQ; +0.5 perfect colocalization, -0.5 perfect anti-colocalization bottom).

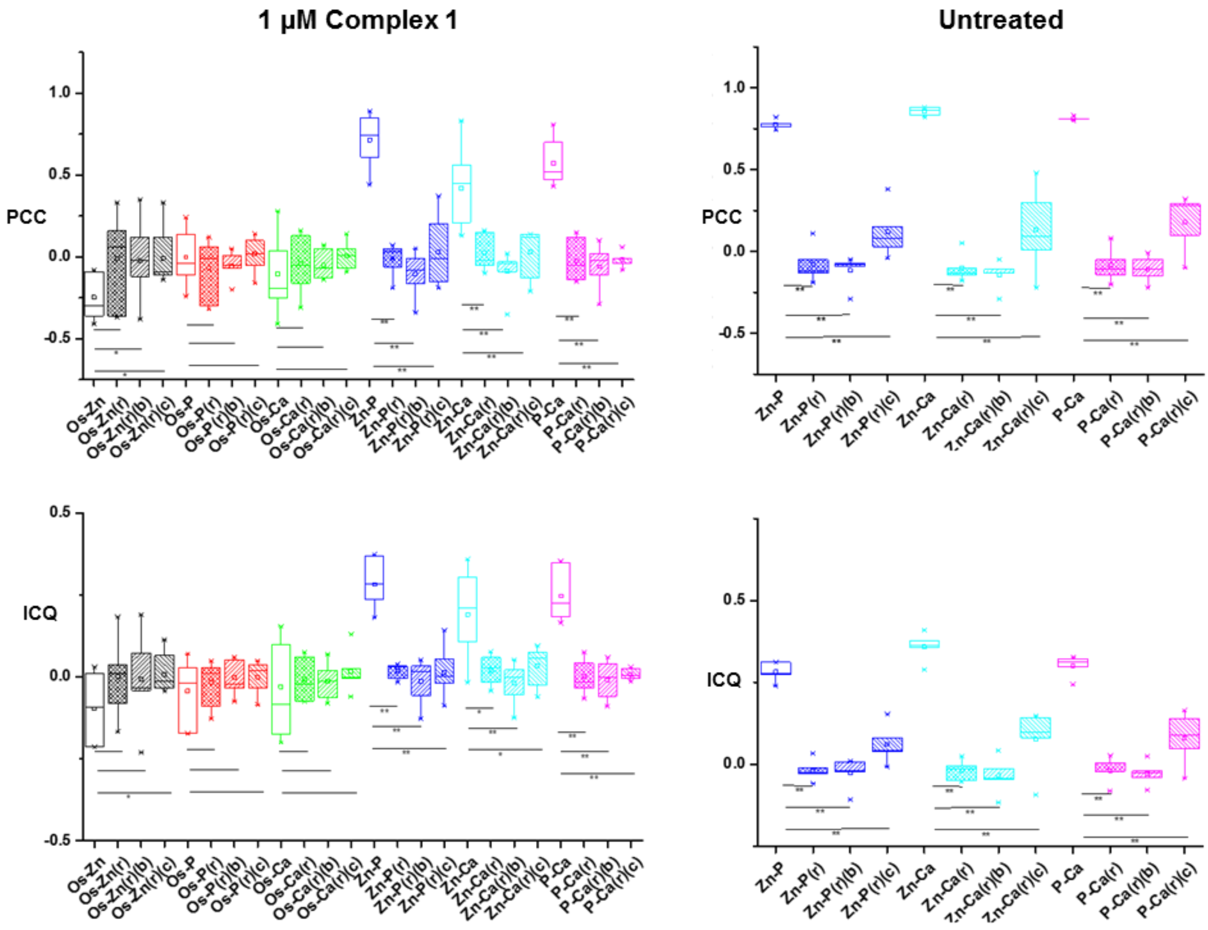

Figure S7. Colocalization analysis for A2780 human ovarian cancer cells treated for 24 h with 1 µM **1** (left)**,** and for untreated cells (right), between different elements of randomly scrambled SXRFN maps (3 different tests) using Pearson's correlation coefficient (PCC; top; +1 perfect colocalization, -1 perfect anti-colocalization; top) and intensity correlation quotient (ICQ; bottom; +0.5 perfect colocalization, -0.5 perfect anti-colocalization bottom).

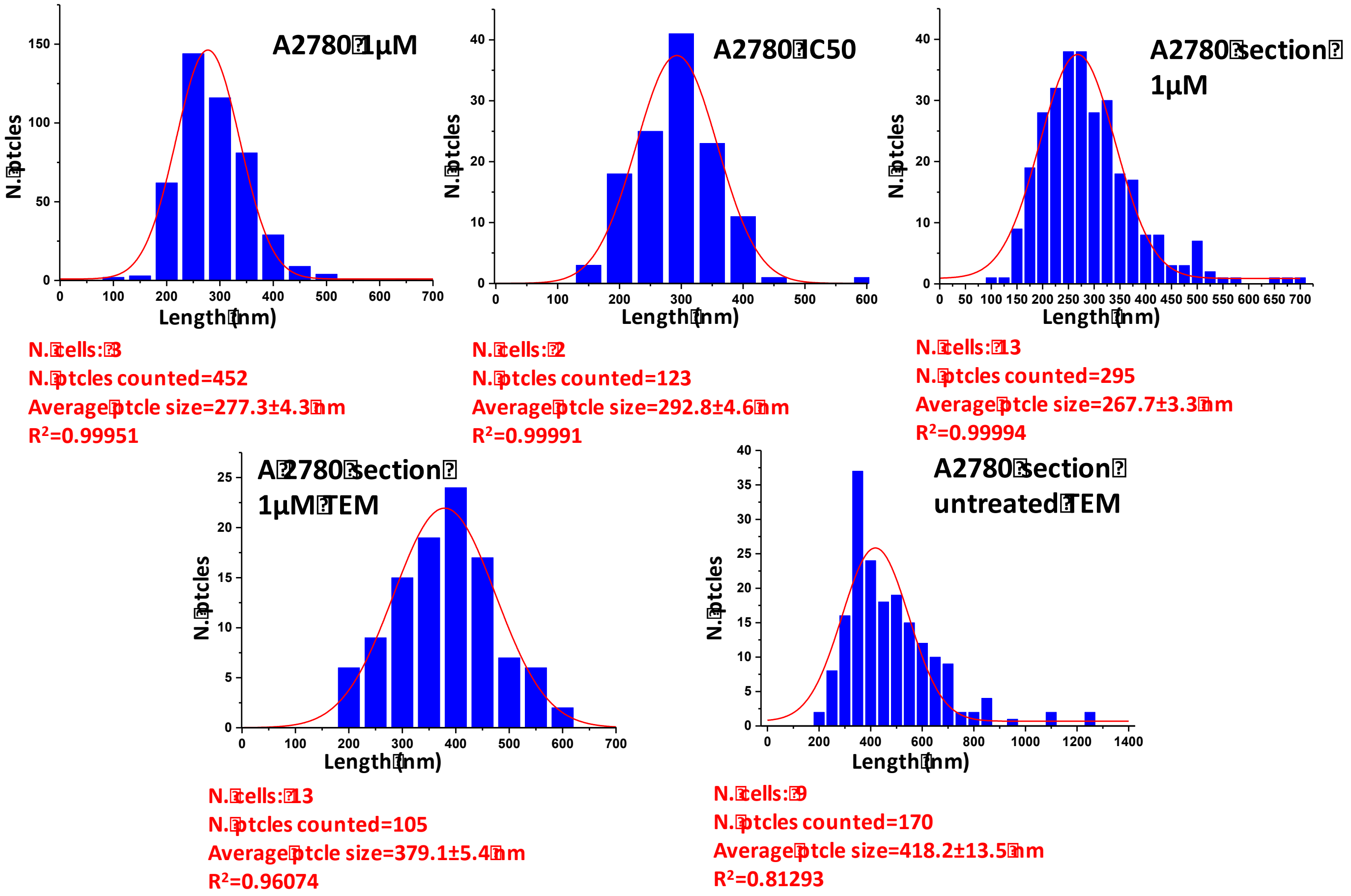

Figure S8. Plots of the size distribution (in nm) of areas with a high density of Os in SXRFN maps from whole A2780 ovarian cancer cells and sections of A2780 cells treated with IC_{50} or 1µM of **1 for** 24 h (Top row), and dark particles in TEM images from whole A2780 cells treated with 0 or 1 µM of **1 for** 24 h (Bottom row). Text below the plots indicates the number of cells analysed and ROIs measured, average size of the ROIs, and R^2 of the fitting Gaussian.

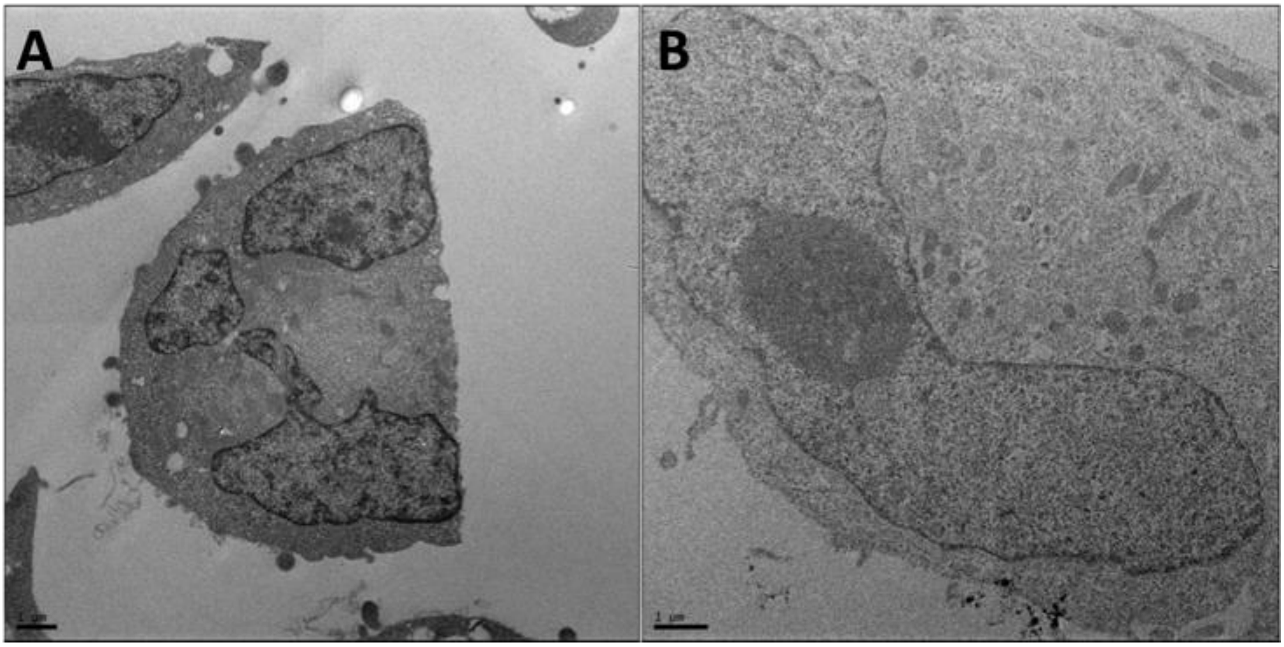

Figure S9. TEM images of stained 100 nm thick sections of A2780 cells A) treated with 1 µM **1** for 24 h, and B) untreated. Scale bar 1 µm.

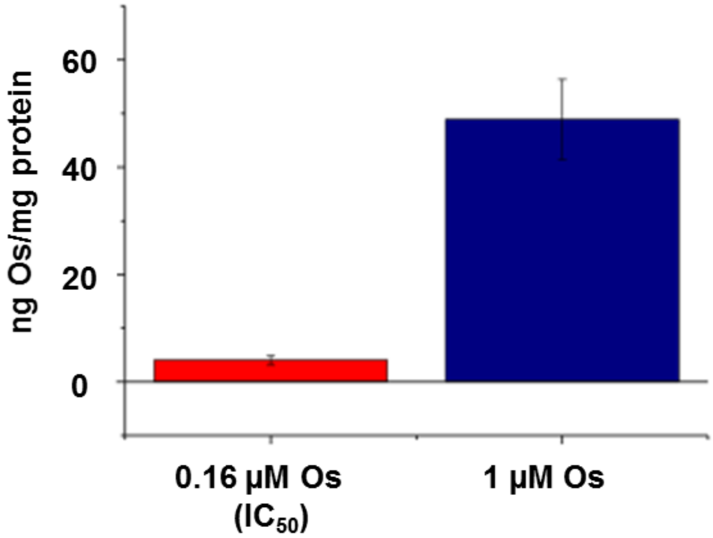

Figure S10. Os found in mitochondrial fractions isolated from A2780 cells treated with IC_{50} or 1 µM concentrations of **1** (in ng/mg of protein).

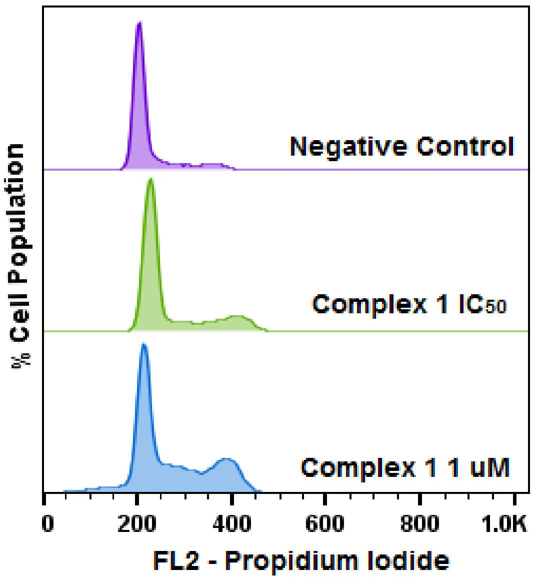

	% Cell Population		
	Control	IC_{50}	1 µM
**Sub G1 Phase**	0.5 ±0.3	2.2 ± 0.5 *	8.7 ± 0.6 **
**G1 phase**	64 ± 2	70.6 ± 0.6 *	42 ± 2 **
**S Phase**	18 ± 2	19.0 ± 0.4	27.7 ± 0.7 **
**G2/M Phase**	17.8 ± 0.2	8.2 ± 0.6 **	21.7 ± 0.8 *

Figure S11. Cell cycle analysis of A2780 cells treated with 0, IC_{50} and 1 µM of complex **1** for 24 h. All values compared to the untreated controls for statistical significance calculations. In all cases, independent two-sample t-tests with unequal variances, Welch's tests, were used ($p < 0.01$ for **, and $p < 0.05$ for *).

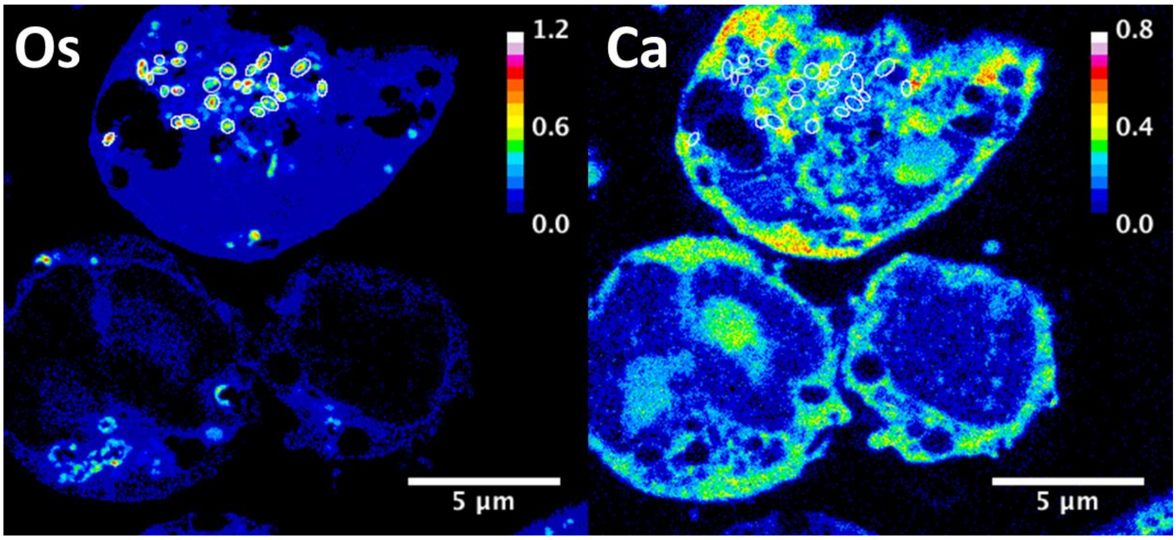

Figure S12. XRF maps of a 500 nm thick section of A2780 cells treated with 1 µM **1** for 24 h showing the cellular distribution of Os and Ca; Raster scan: 50x50 nm^2, 50 ms dwell time. White ellipses show the absence of Ca in areas with a high density of Os. Scale bar 5 µm. Calibration bar in ng/mm^2.
